# Practical First Principles

**Published:** 1901-10

**Authors:** 


### *BOOK REVIEWS, PAMPHLETS, EXCHANGES.*

---

PRACTICAL FIRST PRINCIPLES. Simplifying the study of normal and abnormal structure and function, and aiding diagnosis. Designed for the use of students and practitioners of medicine. By A. H. P. Leuf, M. D., associate editor of *The Medical Council,* Philadelphia. Published by The Medical Council, Twelfth and Walnut Sts. 8 vo., 105 pages, nearly 50 illustrations, almost all new and original. Price $1.00 net.

This work is original in conception, is true, and recognizes the detached mode of instruction now and always in vogue in the teaching of medicine, on which account it has been offered to the pro-ession to make up this deficiency by supplying the connecting links between the various departments. To the student beginning the study of medicine, it gives a comprehensive resume of some of the most important principles which he has to continually apply, easing his understanding and greatly simplifying his studies. It teaches underlying principles and their general application, so that a fact learned of one tissue applies equally to all. Thus inflammatory processes are divided into acute or chronic, and parenchymatous and interstitial. Once the easily remembered facts of these inflammatory changes are understood of one tissue, they become applicable to all, so that if the histologic structure and the function of a part are known, its significance and symptomatology may be quite accurately inferred.

The book is the nearest approach to a royal road to knowledge that we have ever encountered. It eases the work of the student and simplifies that of the practicing physician. It is to be read and reread, studied, digested and absorbed.

Beginning with protoplasm and the cell, it proceeds in sequence to the consideration of tissues, organs, apparatuses, systems, the body as a whole, cell nutrition, cell physiology, cell pathology, general principles, their illustrative application, nomenclature, and an appendix dealing with an explanation of the microscope.

There are few men who do not need this book and none who can regret having it, provided they are true students and conscientious practitioners. To all such it will prove, as it was intended to be, a source of pleasure and of profit.

